# A case of acute pancreatitis caused by obstruction of the pancreatic duct with a plant-based broom bristle

**DOI:** 10.1055/a-2689-3319

**Published:** 2025-09-04

**Authors:** Tan Chen, Shuhui Zhang, Mingwei Fan, Aili Wang, Yan Chen, Xianyong Cheng

**Affiliations:** 1 562131 Institute of Digestive Diseases, Binzhou Medical University Hospital, Binzhou, China; 2 694180 Department of Gastroenterology, Yangquan First Peopleʼs Hospital, Yangquan, China; 3 562131 Department of Gastroenterology, Binzhou Medical University Hospital, Binzhou, China

Acute pancreatitis is an inflammatory disease causing acute abdominal pain. Most cases are mild, but 15%–20% may develop complications leading to organ failure and mortality [1]. Causes include metabolic issues, injury, drugs, ischemia, infection, and genetic damage [1]. Biliary and pancreatic duct obstruction is a common factor.

Biliopancreatic duct obstruction can result from stones, inflammation, tumors, congenital abnormalities, and other diseases [1]. Foreign body-induced obstruction is rare. Here, we report a case where a foreign body in the pancreatic duct caused acute pancreatitis.

A 57-year-old woman was referred to our hospital for evaluation and management of acute pancreatitis of unknown etiology. After the patient was admitted to the hospital, laboratory tests, upper abdominal ultrasound, and enhanced computed tomography (CT) scans of the patient all indicated acute pancreatitis.

After five days of routine treatment, the patient showed no significant improvement. The patient was recommended to undergo gastroscopy and endoscopic ultrasound to identify the cause of the pancreatitis. Gastroscopy revealed purulent secretions and a foreign body in the duodenal papilla ( Fig. 1 ). Endoscopic ultrasound (EUS) detected high echogenicity in the main pancreatic duct, suggesting a foreign body ( Fig. 2 ). The foreign body was successfully grasped and extracted using biopsy forceps ( Video 1 ).

**Fig. 1:**
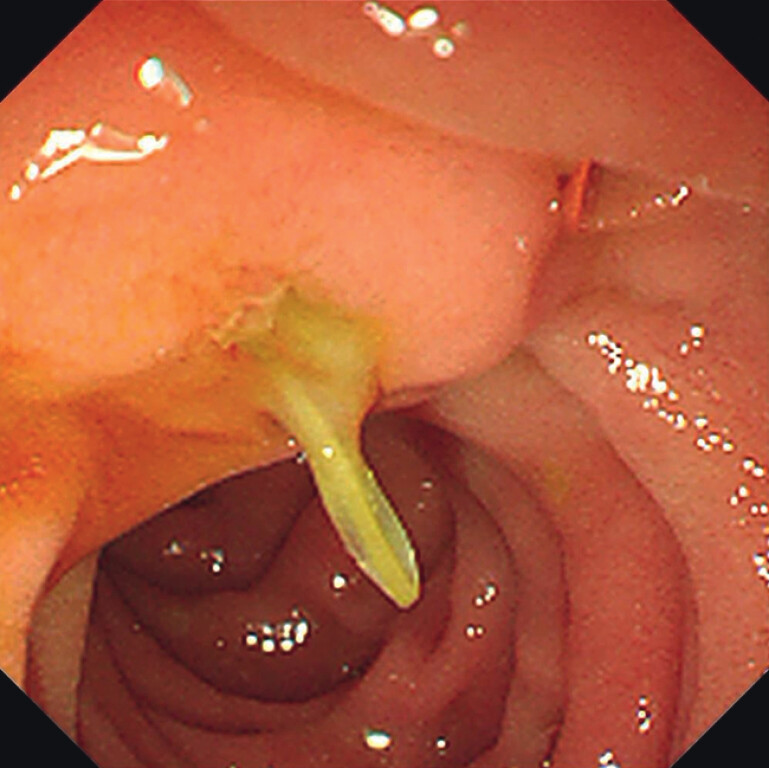
Endoscopic images depicted a foreign body entrapped in Vater's papilla, with protrusion into the lumen of the duodenum.

**Fig. 2:**
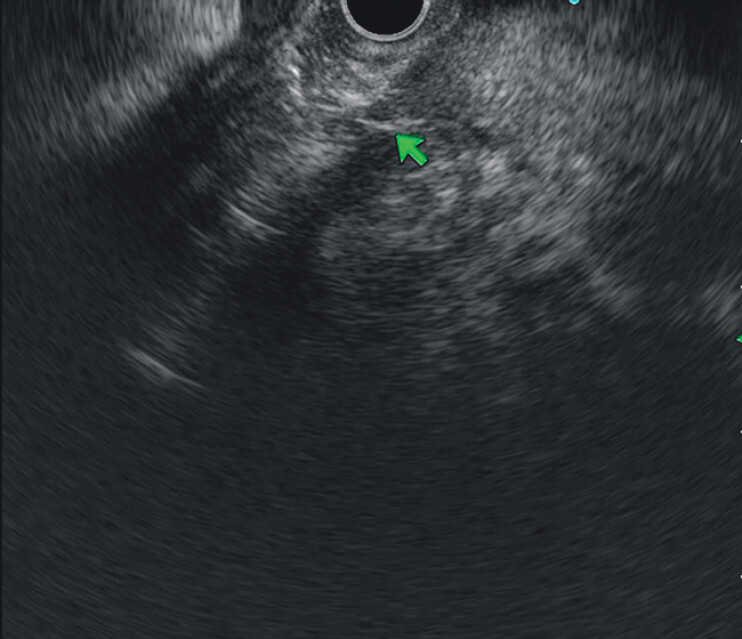
Ultrasound endoscopic image. Before foreign body removal (the green arrow points to the foreign body).

Video of endoscopic removal of gallbladder and pancreatic duct foreign bodies.Video 1

After the foreign body was removed, a repeat EUS showed the disappearance of the hyperechoic shadow in the main pancreatic duct, with mild wall roughness and no significant dilation ( Fig. 3 ). The extracted foreign body was identified as a 5.5-cm plant-based broom bristle ( Fig. 4 ).

**Fig. 3:**
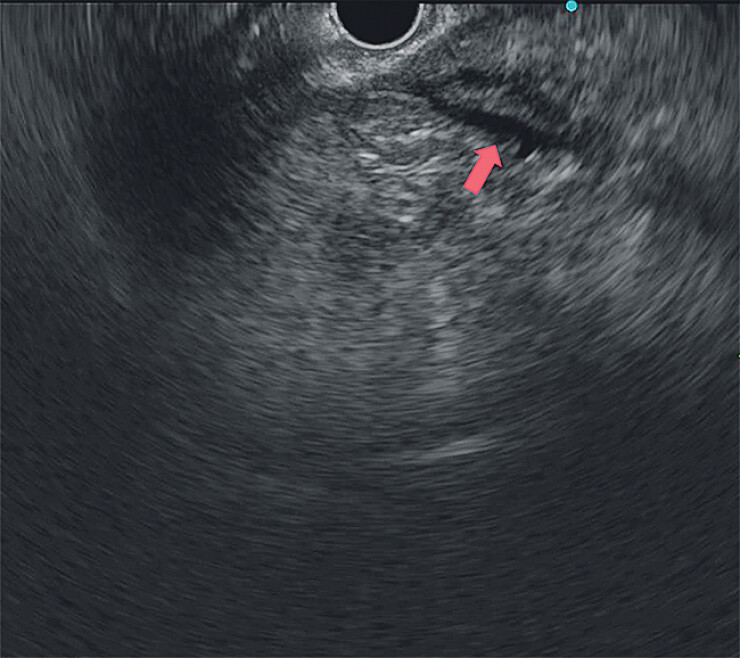
Ultrasound endoscopic image. After foreign body removal (the red arrow points to the main pancreatic duct).

**Fig. 4:**
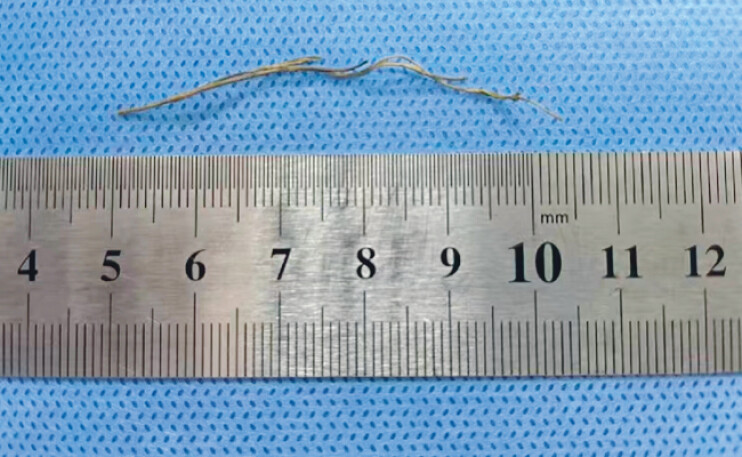
Photo of the foreign body.

The patient improved after surgery, denied any history of foreign body ingestion when questioned, and continued with anti-inflammatory therapy. She was discharged three days later after clinical improvement and remained relapse-free during follow-up.

The occurrence of pancreatitis due to pancreatic duct obstruction is rare. CT scans usually detect foreign bodies accurately, but missed diagnoses can happen in specific cases 4 . In this case, neither CT nor ultrasound revealed foreign bodies, and the patient did not report ingestion history. Further EUS examination identified a foreign body as the cause of pancreatitis. Physicians should remain vigilant and use appropriate diagnostic methods for complex cases.

Endoscopy_UCTN_Code_CCL_1AB_2AF

## Notes

Endoscopy E-Videos https://eref.thieme.de/e-videos
*E-Videos* is an open access online section of the journal *Endoscopy* , reporting on interesting cases and new techniques in gastroenterological endoscopy. All papers include a high-quality video and are published with a Creative Commons CC-BY license. Endoscopy E-Videos qualify for HINARI discounts and waivers and eligibility is automatically checked during the submission process. We grant 100% waivers to articles whose corresponding authors are based in Group A countries and 50% waivers to those who are based in Group B countries as classified by Research4Life (see: https://www.research4life.org/access/eligibility/ ). This section has its own submission website at https://mc.manuscriptcentral.com/e-videos .
